# Serum uri acid: neuroprotection in thrombolysis. The Bergen NORSTROKE study

**DOI:** 10.1186/1471-2377-11-114

**Published:** 2011-09-25

**Authors:** Nicola Logallo, Halvor Naess, Titto T Idicula, Jan Brogger, Ulrike Waje-Andreassen, Lars Thomassen

**Affiliations:** 1 Department of Neurology, Haukeland University Hospital, Bergen, Norway; 2 Department of Clinical Medicine, University of Bergen, Bergen, Norway

## Abstract

**Background:**

A possible synergic role of serum uric acid (SUA) with thrombolytic therapies is controversial and needs further investigations. We therefore evaluated association of admission SUA with clinical improvement and clinical outcome in patients receiving rt-PA, early admitted patients not receiving rt-PA, and patients admitted after time window for rt-PA.

**Methods:**

SUA levels were obtained at admission and categorized as low, middle and high, based on 33° and 66° percentile values. Patients were categorized as patients admitted within 3 hours of symptom onset receiving rt-PA (rt-PA group), patients admitted within 3 hours of symptom onset not receiving rt-PA (non-rt-PA group), and patients admitted after time window for rt-PA (late group). Short-term clinical improvement was defined as the difference between NIHSS on admission minus NIHSS day 7. Favorable outcome was defined as mRS 0 - 3 and unfavorable outcome as mRS 4 - 6.

**Results:**

SUA measurements were available in 1136 patients. Clinical improvement was significantly higher in patients with high SUA levels at admission. After adjustment for possible confounders, SUA level showed a positive correlation with clinical improvement (r = 0.012, 95% CI 0.002-0.022, p = 0.02) and was an independent predictor for favorable stroke outcome (OR 1.004; 95% CI 1.0002-1.009; p = 0.04) only in the rt-PA group.

**Conclusions:**

SUA may not be neuroprotective alone, but may provide a beneficial effect in patients receiving thrombolysis.

## Background

Serum uric acid (SUA) is a final enzymatic product of purine metabolism [1,2]. Animal models of acute ischemic stroke (AIS) have shown that SUA may be neuroprotective [3] and may reinforce the benefits of intravenous thrombolysis (rt-PA) [4]. In humans, high SUA may be an independent predictor of better outcome after AIS [5] and represent a consumptive and reproducible antioxidant in AIS [6,7]. Combined intravenous administration of rt-PA and SUA is safe, prevents an early decline in SUA levels and decreases lipid peroxidation but has not shown any clinical effect [8]. On the other hand, high SUA levels have been also associated with hypertension [9], dyslipidemia, type 2 diabetes [10], kidney disease, cardiovascular and cerebrovascular events [11] and worse functional outcome after AIS [12]. The role of SUA in AIS is therefore still controversial, and a possible synergic role of SUA with thrombolytic therapies needs further investigations.

In the present study we therefore aimed to evaluate the association of admission SUA levels with short-term clinical improvement and short-term clinical outcome in patients receiving rt-PA, patients admitted within 3 hours of symptom onset not receiving rt-PA, and patients admitted after the time window for rt-PA.

## Methods

This prospective study was conducted at Haukeland University Hospital, Bergen, Norway, which serves a well-defined population of 235,000 inhabitants. The study was performed from February 2006 to January 2009 as a part of a cohort study (Bergen NORSTROKE Study) in which data are collected from all consecutive ischemic stroke patients admitted to the stroke unit in the Department of Neurology. All patients were managed according to a standard protocol and received standard care as recommended by the European Stroke Organisation [13]. Eligible patients received intravenous rt-PA according to the SITS-MOST protocol [14]. SUA levels, NIH stroke scale (NIHSS) score, serum creatinine levels, systolic and diastolic blood pressure were obtained at admission. Patients with unavailable SUA levels at admission were excluded from the study. Patients were categorized as patients admitted within 3 hours of symptom onset receiving rt-PA (rt-PA group), patients admitted within 3 hours of symptom onset not receiving rt-PA (non-rt-PA group), and patients admitted after time window for rt-PA (late group). Patients were categorized as young (≤ 65 years) and old (> 65 years). NIHSS scores were categorized as mild (< 8), moderate (8 - 14) and severe (> 14) [15]. Short-term clinical progress was defined as the difference between NIHSS on admission (NIHSS0) minus NIHSS at day 7 (NIHSS7) (Δ-NIHSS = NIHSS0 - NIHSS7) [16,17]. A positive Δ-NIHSS value indicated a clinical improvement whereas a negative value indicated a clinical worsening.

SUA were categorized as low, middle and high, based on 33° and 66° percentile values.

The etiology of stroke was classified as large-artery atherosclerosis, cardioembolism, small vessel occlusion, stroke of other determined etiology and stroke of undetermined etiology based on TOAST criteria [18].

Diabetes mellitus (DM) was defined as treatment with glucose lowering medications or diet before stroke onset. Hypertension (HT) was defined as treatment with antihypertensive drugs before stroke onset. Serum creatinine levels were measured at admission and categorized as normal < 120 μmol/L or high ≥ 120 μmol/L. Previous stroke or TIA were registered. Seven days after stroke onset, functional outcome was assessed by modified Rankin scale (mRS) [19]. Assessment was done by a trained stroke nurse. Favorable outcome was defined as mRS 0 - 3 and unfavorable outcome as mRS 4 - 6 [20]. The study was approved by the local research ethics committee and informed consent was obtained from all patients as part of a prospective study protocol.

### Statistics

Chi-square test, Student's t-test, and analysis of variance (ANOVA) were used as appropriate. Age, sex, systolic blood pressure at admission, serum creatinine, pre-existing diabetes were considered as possible confounding factors of SUA level. Multiple linear regression was used to estimate the relationship between SUA and Δ-NIHSS. Age, sex, systolic blood pressure at admission, serum creatinine, pre-existing diabetes and admission NIHSS score were included as other exposure variables. Logistic regression analysis was performed to determine factors that could be considered independent predictors for stroke outcome. Significance was set at p < 0.05. The analysis was performed with the software 'STATA/SE 11.0 for Windows'.

## Results

During the study period, 1224 patients with ischemic stroke were admitted to our department. SUA measurements were available in 1136 patients. Missing values were due to technical error and may be called protocol violations. Basic characteristics of the study population are shown in Table 1. SUA levels related to demographics and risk factors of the population groups are shown in Table 2.

**Table 1:** Basic Characteristics of the Study Population (n = 1136)

	rt-PA	Non rt-PA	Late	p
Sample size, n	186	261	689	
Time to blood sample, h (IR)	1.5 (1.0-2.4)	1.9 (1.4-2.7)	18.7 (9.3-31.1)	
Age, years (SD)	69.3 (± 14.2)	70.9 (± 14.1)	71.4 (± 14.6)	0.21
Male Sex, n (%)	118 (63.4)	149 (57.1)	385 (± 55.9)	0.17
Median NIHSS day 0* (IR)	9.5 (4-17)	3 (1-5)	3 (1-7)	0.0001
NIHSS day 0, n (%)				0.0001
< 8	76 (40.9)	216 (82.7)	524 (76.1)	
8-14	42 (22.6)	13 (7.3)	78 (11.3)	
> 14	68 (36.5)	26 (10.0)	87 (12.6)	
SUA levels, μmol/L, n (%)				0.2
< 305	56 (29.5)	93 (34.7)	257 (33.5)	
305-399	63 (33.2)	100 (37.3)	249 (32.5)	
≥ 400	71 (37.4)	75 (28.0)	260 (34.0)	
Creatinine, μmol/L, n (%)				0.4
< 120	169 (91.0)	247 (92.1)	687 (89.7)	
≥ 120	17 (9.0)	21 (7.9)	79 (10.3)	
SBP day 0, mmHg (SD)	162.0 (± 25.9)	168.5 (± 29.3)	167.5 (± 33.6)	0.05
Previous diseases, n (%)				
Hypertension	92 (49.5)	133 (50.0)	415 (54.6)	0.3
Diabetes	23 (12.4)	33 (12.6)	119 (15.8)	0.3
Stroke	21 (11.0)	58 (21.7)	145 (19.1)	0.01
TIA	18 (9.4)	28 (10.5)	71 (9.3)	0.8
Median mRS day 7 (IR)	2 (1-4)	2 (1-3)	2 (1-4)	0.01

IR, Interquantile Range; SD, Standard Deviation; NIHSS, National Institutes of Health Stroke Scale; SUA, serum Uric Acid; SBP, systolic blood pressure; TIA, transient ischemic attack; mRS, modified Rankin Scale

*Day 0: day of admission

**Table 2:** Serum Uric Acid Levels on admission in Relation to Demographics and Risk Factors in the population subgroups

	SUA at admission, μmol/L (± SD)			
	rt-PA	Non rt-PA	Late	
	n = 186	n = 261	n = 689	p
Overall	361 (± 97)	348 (± 93)	345 (± 104)	0.03
Age				
(> 65)	367 (± 101)	358 (± 90)	357 (± 113)	0.6
(≤ 65)	352 (± 92)	330 (± 95)	324 (± 82)	0.01
p*	0.1	0.01	0.0001	
Female	331 (± 102)	310 (± 89)	325 (± 111)	0.3
Male	378 (± 90)	377 (± 85)	361 (± 55.9)	0.17
p*	0.0007	0.0001	0.0001	
NIHSS day 0				
< 8	349 (± 86)	347 (± 91)	341 (± 96)	0.6
8-14	359 (± 107)	351 (± 108)	356 (± 109)	0.9
> 14	375 (± 103)	355 (± 93)	362 (± 137)	0.7
p*	0.2	0.9	0.1	
Hypertension				
Yes	396 (± 92)	364 (± 94)	365 (± 105)	0.003
No	327 (± 90)	334 (± 88)	321 (± 96)	0.3
p*	0.0001	0.005	0.0001	
Diabetes				
Yes	372 (± 109)	361 (± 88)	361 (± 113)	0.3
No	360 (± 96)	347 (± 93)	341 (± 101)	0.03
p*	0.3	0.2	0.03	

SUA, serum Uric Acid; SD, Standard Deviation; NIHSS, National Institutes of Health Stroke Scale.

* p values for statistical tests assessing difference in SUA between categories of each population subgroup.

**Figure 1:**
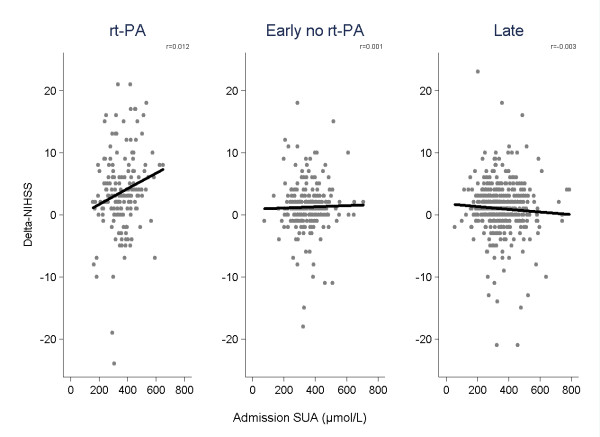
**Correlation between SUA levels and Δ-NIHSS**. Age, gender, NIHSS0, serum creatinine and admission systolic blood pressure are included as other exposure variables in the multiple linear regression model. Δ-NIHSS: NIHSS on admission (NIHSS0) minus NIHSS at day 7 (NIHSS7) rt-PA: r = 0.012, 95% CI 0.002-0.022, p = 0.02 Early, no rt-PA: r = 0.012, 95% CI -0.004-0.007, p = 0.7 Late: r = -0.003, 95% CI -0.006-0.0002, p = 0.07.

Among the three groups, the rt-PA group had the largest short-term clinical improvement (Table 1). Within this group, the improvement was significantly larger in patients with high SUA levels at admission (Table 3). Multiple linear regression that included patients characteristics (age, gender), stroke characteristics (NIHSS score at admission) and possible confounding factors for SUA levels (serum creatinine, systolic blood pressure at admission) as other exposure variables, showed a positive correlation between admission SUA levels and Δ-NIHSS only in the rt-PA group (r = 0.012, 95% CI 0.002-0.022, p = 0.02), Figure 1.

**Table 3:** Early Clinical Progress (*Δ-NIHSS) and SUA levels (μmol/L) in the three population subgroups

		Δ-NIHSS (± SD)				
	**SUA levels**	**All**	**< 305**	**305-399**	**≥ 400**	**p**

rt-PA group		3.62 (± 6.3)	3.0 (± 6.2)	2.2 (± 6.6)	5.2 (± 5.9)	< 0.01
Early, no rt-PA		1.2 (± 4.0)	1.4 (± 3.8)	0.8 (± 4.2)	1.3 (± 4.6)	0.2
Late group		1.0 (± 3.7)	1.3 (± 3.1)	0.9 (± 5.2)	1.0 (± 4.4)	0.3

*Δ-NIHSS: NIHSS on admission (NIHSS0) minus NIHSS at day 7 (NIHSS7)

Stepwise logistic regression analysis including the same covariates as the multiple linear regression, identified SUA levels as predictor for favorable stroke outcome (mRS 0-3) only in the rt-PA group (OR 1.004; 95% CI 1.0002-1.009; p = 0.04). Young age and low NIHSS score at admission were predictors for better stroke outcome in all the three groups.

## Discussion

To be effective, neuroprotective agents should act in the acute phase of ischemic stroke, before neuronal death has occurred. We therefore focused our study on SUA levels measured within the first 3 hours after stroke onset. Antioxidant agents have so far shown to be effective only in animal models of AIS, but not in humans [21]. Potential factors hindering translation of animal models success into clinical practice may be patient heterogeneity, inappropriate dose, and wide time window [22]. SUA is a natural antioxidant present in biological fluids throughout the body, and appears to be the major endogenous antioxidant [23,24]. However, it has been controversial whether SUA is neuroprotective or neurotoxic. Recently, it has been proposed that SUA may show both anti- and pro-oxidant properties depending on levels of other antioxidants, levels of oxidative stress and time of interaction with the target tissues [25], and that the balance between the anti- and pro-oxidant properties shifts in favour of neuroprotection in conditions of extraordinary oxidative stress such as AIS [24]. In our study, SUA level did not show any correlation with clinical improvement nor association with better clinical outcome in early patients not receiving rt-PA. Early studies investigating a possible association between SUA and clinical outcome showed controversial results [5,12]. However, a potential limitation of these studies may be the wide time window for SUA measurements (e.g up to 48 and 72 hours). Moreover, the study which showed that SUA level predicts poor outcome after AIS [12] included also patients with TIA and intracranial hemorrhage, and did not specify whether a percentage of AIS patients received thrombolytic therapy.

Thrombolysis may lead to recanalization which can salvage viable tissue in the penumbral zone, and is currently the only approved treatment for acute stroke. Dual therapy with rt-PA and neuroprotective agents has shown promising results only in model of AIS [26,27]. Combined administration of uric acid and rt-PA to adult rats twenty minutes after the induction of ischemia caused a significant reduction in infarct volume and a significant lower neurological impairment [3,4]. In humans, higher SUA levels in AIS patients receiving rt-PA have been associated with better outcome at day 90 and smaller infarct volume [28]. In agreement with this, our study shows that high SUA is independently associated with higher short-term clinical improvement and better short-term clinical outcome in rt-PA patients.

During the first hours of AIS, reactive oxygen species (ROS) produced by mitochondria play a central pathogenetic role in neuronal death. A further burst of ROS occurs during the early tissue reperfusion induced by recanalization [29]. Serial SUA measurements have shown that SUA is consumed during the first six hours after stroke [6,7], suggesting that SUA represents a consumptive and reproducible antioxidant in AIS. SUA may therefore supply an additional beneficial effect to rt-PA by acting as a scavenger engulfing ROS released during the early recanalization induced by rt-PA. Even though we do not have data on recanalization rate, this is likely to be higher in the rt-PA group than in the early non-rt-PA group.

## Conclusion

In our study high SUA was independently associated with higher short-term clinical improvement and better short-term clinical outcome in rt-PA patients whereas no association was found in early patients not receiving rt-PA or in late patients. SUA may not achieve clinical effect alone, but may provide an additional beneficial effect in patients receiving recanalizing therapies.

## Competing interests

The authors declare that they have no competing interests.

## Authors' contributions

NL was involved in the conception and design of the study, acquisition, analysis and interpretation of data, and drafting the manuscript. HN was involved in the conception and design of the study, and acquisition, analysis and interpretation of data. JB was involved in the conception and design of the study, and acquisition, analysis and interpretation of data. TTI was involved in acquisition of data and drafting the manuscript. UWA was involved in the conception and design of the study, acquisition, analysis and interpretation of data and drafting the manuscript. LT was involved in the conception and design of the study, acquisition, analysis and interpretation of data, and drafting the manuscript. All authors participated in the revision of the manuscript for important intellectual content and approved the final manuscript.

## Pre-publication history

The pre-publication history for this paper can be accessed here:

http://www.biomedcentral.com/1471-2377/11/114/prepub
